# Patient Participation and the Environment: A Scoping Review of Instruments

**DOI:** 10.3390/ijerph19042003

**Published:** 2022-02-11

**Authors:** Maya Kylén, Ulla-Karin Schön, Hélène Pessah-Rasmussen, Marie Elf

**Affiliations:** 1 School of Health and Welfare, Dalarna University, 791 88 Falun, Sweden; ullakarin.schon@socarb.su.se (U.-K.S.); mel@du.se (M.E.); 2 Department of Health Sciences, Lund University, 221 00 Lund, Sweden; helene.pessah@skane.se; 3 Department of Social Work, Stockholm University, 106 91 Stockholm, Sweden; 4 Department of Neurology, Rehabilitation Medicine, Memory Disorders and Geriatrics, Skåne University Hospital, 221 85 Lund, Sweden; 5 Department of Clinical Sciences, Lund University, 221 84 Lund, Sweden

**Keywords:** patient participation, healthcare, environment, scoping review, patient-reported instruments

## Abstract

Patient participation and the environment are critical factors in achieving qualitative healthcare. We conducted a systematic scoping review using Arksey and O’Malley’s framework to identify instruments intended to measure patient participation. We assessed those instruments’ characteristics, which areas of the healthcare continuum they target, and whether environmental factors are considered. Instruments were considered eligible if they represented the patient perspective and measured patient participation in healthcare. The search was limited to articles written in English and published in the last 10 years. We extracted concepts (i.e., patient empowerment, patient participation, and patient-centeredness) based on the framework developed by Castro et al. and outcomes of significance regarding the review questions and specific objectives. The search was conducted in PsycINFO, CINHAL/EBSCO, and PubMed in September 2019 and July 2020. Of 4802 potential titles, 67 studies reported on a total of 45 instruments that met the inclusion criteria for this review. The concept of patient participation was represented most often in these studies. Although some considered the social environment, no instrument was found to incorporate and address the physical environment. Thirteen instruments were generic and the remaining instruments were intended for specific diagnoses or healthcare contexts. Our work is the first to study instruments from this perspective, and we conclude that there is a lack of instruments that measure aspects of the social and physical environment coherently as part of patient participation.

## 1. Introduction

Patient participation is a key component of person-centered care and the quality of health and social care [1,2,3]. In person-centered care, a service must provide patients with greater decision-making power and more choices and integrate the environment and patients’ unique physical, psychosocial, cultural, and emotional needs [4]. Patient participation is associated with better healthcare processes and patient health outcomes, reduced mortality, and lower healthcare costs [5,6,7]. Patient participation has also been shown to improve motivation, treatment commitment, and self-management among persons living with chronic conditions [8,9,10]. Current healthcare services use a range of strategies to evaluate the participation of patients and their significant others which are important for the purpose of continuous improvements [11]. For example, the questionnaire Quality from the Patient’s Perspective [12] has been used to measure the quality of care by asking questions about patients’ participation experiences in surgical units [13]. However, despite its growing significance, patient participation has been poorly implemented in practice and the concept has been vaguely defined [14].

Alongside patient participation, the environment is a key factor in attaining quality healthcare and supporting patient health outcomes [15]. Environmental factors are placed at the center in many rehabilitation and disability health models [15,16,17]. However, little is known about how to integrate environmental factors in the quest to improve care quality and patient health outcomes [18,19]. As disability is created when a person with an injury or health condition interacts with an environment that is not supportive [20], this lack of knowledge is a concern. For persons with disabilities, environmental factors have a major impact on everyday participation at the immediate, community, and societal levels [21]. This can be exemplified by the many patients who are discharged from the hospital to homes that are not supportive of their new health situation which may result in activity and participation restrictions as well as being unsafe [22]. Thus, the current trend of short hospital stays for persons with complex health conditions and continued rehabilitation and care in the home [23,24] calls for greater inclusion of the environment in health-related communication across the continuum of care.

The environment comprises a multitude of factors that can either be a barrier to or support patients’ participation, functioning, and right to preserve their personal integrity [15,17]. For example, in the International Classification of Functioning, Disability, and Health (ICF), environmental factors are defined as those that “make up the physical, social and attitudinal environment in which people live and conduct their lives” [16]. Physical features include the built environment (e.g., stairs or doors), natural environment (e.g., outdoor surfaces), and objects, whereas the social environment can be defined to include the persons with whom one interacts, supports, and has relationships [16]. The impact the environment may have on an individual’s life and their possibilities to function depend on the degree of support (e.g., practical, emotional) or demand (e.g., accessibility, usability) that the physical and social environment may have [17]. For example, person-environment fit theories describe that the adequacy of the fit between a person’s functional abilities and environment can affect the person’s level of independence, participation, and overall health and well-being [25]. If the fit is good, the person’s functioning may be facilitated, and if incompatible, the person may experience maladaptation [26]. Acknowledged also by the World Health Organization (WHO), the ICF states that environmental factors and participation are both crucial for patient health outcomes [16]. Hence, healthcare professionals must integrate environmental factors into their assessments, goal setting, and evaluations to promote patient participation.

When a patient is informed rather than involved, there is a risk that important information about the patient´s environmental prerequisites will be lost [27]. For example, inadequate practices of discharge from the hospital—that is, discharge that does not include a dialogue with the patient about his or her home environment and needs post-discharge—may have adverse effects such as dependency in daily activities and social isolation [22]. In addition, the main goal of persons living with complex health conditions is to be part of society. Despite this, much of today’s healthcare concentrates on helping patients restore their functional capacity and improve their independence in daily life, while goals involving a person’s activities in the environment outside the home are seldomly addressed [28,29,30,31].

The concept of patient participation has been poorly defined, and various synonymous terms, such as patient involvement, empowerment, patient-centeredness, and person-centered care, have been used interchangeably in the literature [14]. However, the increasing emphasis on participation by the WHO, governments, patient organizations, and health and social care policies makes it important that we understand the concept of how to measure patient participation and what enables it across the continuum of care. 

From an environmental perspective, two things are important for patient participation. First, clinical routines and attitudes need to enable patients to influence and engage in care decisions. Secondly, care interventions should be adapted to the patient’s personal preferences and allow patients to participate in everyday life in society [32,33]. We use the framework of Castro and colleagues who define patient participation as revolving around a patient’s rights and opportunities to influence and engage in decision making about his or her care through a dialogue attuned to her or his preferences, potential, and a combination of her or his experiential and professional expert knowledge [32]. Hence, patient participation addresses the relationship between patients and clinicians within healthcare systems, but it is also a practice with a common goal to include the patient in the healthcare system [14]. Castro argued that patient participation can be seen as an approach to achieve person-centered care and promote patient empowerment [32]. Thus, patient participation includes three essential related concepts: patient empowerment, patient participation, and patient-centeredness. Although several related systematic reviews have been published, patient participation is an evolving concept demanding repetitive re-evaluations [5,34,35,36], especially regarding the role of the environment.

In summary, patient participation is high on the political agenda and considered important within the scientific community, but the concept remains unexplored, especially in regard to the role played by the environment. To guide researchers and, in particular, healthcare professionals in their choice of an appropriate instrument for patient participation and to optimize existing instruments, knowledge of how environmental factors are represented is needed. In this work, we included self-reported instruments (i.e., the respondents read the question and give their responses by themselves without interference) to measure patient participation for specific health conditions and care contexts. This strategy made it possible to identify the instruments that cover environmental aspects and the generic instruments that can be used in different contexts which we argue is essential for empowering patients in person-centered care. The objective of this review was to describe the available instruments for measuring patient participation, evaluate each instrument for the presence of environmental factors, and investigate the area of the healthcare continuum at which they are targeted.

## 2. Materials and Methods

A scoping review methodology based on the framework of Arksey and O’Malley and recommendations by Levac et al. was used to synthesize the research and map the nature, volume, and characteristics of research within a field of interest [37,38]. The preferred reporting items for systematic reviews and meta-analyses extension for scoping reviews (PRISMA-ScR) checklist was used to ensure that all relevant aspects of scoping reviews were included [39]. The following research questions were developed.

- What are the available instruments used for measuring the aspects of patient participation in healthcare?
- What aspects of patient participation do these instruments address?
- Which areas of the healthcare continuum do these instruments target?
- Which environmental aspects do these available instruments include?

The systematic process to answer these questions involved the following phases: the identification of the research question, the identification of relevant studies, study selection, the charting of the data, and the collating, summarizing, and reporting of the results.

### 2.1. Identifying Studies

The initial search was conducted on 2 September 2019, in PsycINFO (psychological, social, behavioral, and health sciences), CINHAL/EBSCO (nursing and allied health), and PubMed (biomedical). The following keywords were used: patient participation, patient involvement, professional-patient relations, client-centered participation, patient empowerment, patient-centered care, patient centeredness, and shared decision making. The following search terms were also used: scale, instrument, and measurement. The search query was tailored to the specific requirements of each database (Supplementary). Qualitative studies were excluded, as were those that were not published in the last 10 years.

A snowball technique was adopted in which citations within articles were hand-searched to identify the original source of instruments. A follow-up search of the three electronic databases was conducted on 12 June 2020, to identify any additional instruments published after the initial search (Supplementary).

### 2.2. Citation Management

All references (*n* = 4802) were imported into the bibliographic manager EndNote X9 [40]. All duplicates were removed first automatically and then by hand (*n* = 1038). Title and abstract relevance screening and the data characterization of full-text articles were conducted in EndNote X9.

### 2.3. Eligibility Criteria

**Figure 1:**
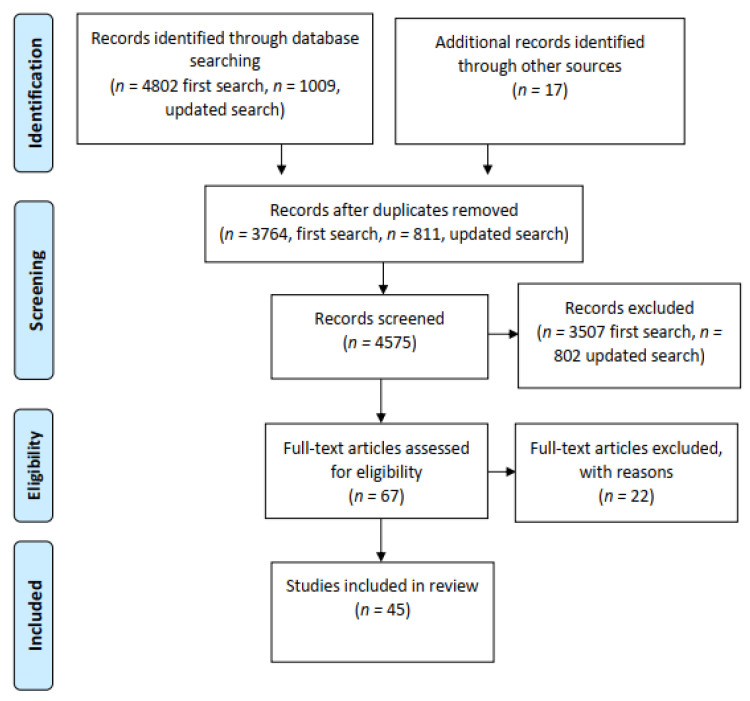
PRISMA chart.

Figure 1 provides a flowchart illustrating the process for screening and assessing the studies identified in the search. After eliminating all duplicates, titles and abstracts were screened for relevance. Studies were eligible for inclusion if they reported on the development and/or validation of questionnaire-based self-reported measurements. The instruments had to represent the patient perspective and measure patient participation in healthcare. Instruments that were not fully developed or validated and studies that included respondents other than patients were excluded.

Articles that did not focus on any of the three essential concepts, patient empowerment, patient participation, and patient-centeredness as defined by Castro [32], were excluded. Additionally, articles that investigated the relationships among variables and used patient participation as an outcome were excluded, but their reference lists were reviewed to identify the original instruments used. When the same instrument was reported in more than one publication (e.g., in a journal article and an electronic report or in a work that has been translated to a different language), only the article reporting the original instrument or modified version was included.

### 2.4. Title and Abstract Relevance Screening

For the first level of screening, only titles and abstracts were reviewed to identify articles that met the inclusion criteria (see Table 1). The title and abstract of each article were independently screened by two reviewers. Titles for which an abstract was not available were included in the subsequent review of full-text articles in the data characterization phase. Reviewers met throughout the screening process to discuss any uncertainties related to study selection [38].

### 2.5. Charting the Data

After title and abstract screening, full-text articles were uploaded to Rayyan, a web-based tool for systematic reviews [41]. If full-text articles were not available, then the authors contacted the respective corresponding author. Each author independently reviewed and labelled all articles and met to resolve any conflicts, ensuring consistency between reviewers and in terms of the research question and purpose [38]. Studies that did not meet the eligibility criteria were excluded.

An Excel file was created and used to extract specific details such as publication year, instrument name, population, terminology, and concepts used to describe patient participation and significant outcomes regarding the review question and specific objectives. Instruments were labeled either as (1) generic, i.e., instruments that could be used for different populations and contexts, or (2) specific, i.e., instruments that could be tested and used in a specific care context (e.g., inpatient care) or for a specific diagnosis (e.g., cancer).

### 2.6. Summarizing and Reporting the Results

The data were coded and validated in Rayyan [41]. At this stage, attention was paid to the domains assessed, the number of items, the theoretical underpinnings, and the presence of environmental factors. The concepts described in the articles based on theories or attributes were reflected in the conceptual framework developed by Castro et al. [32]. However, there remains no consensus on the use and meaning of these concepts, which is why attempts to thematize the relevant studies have been considered valuable.

## 3. Results

The original and updated searches yielded 4802 and 1009 potentially relevant articles, respectively. After deduplication (*n* = 3764 in the first search and 811 in the updated search) and relevance screening, 50 articles met the inclusion criteria based on titles and abstracts, and the corresponding full-text articles were thus reviewed. In addition, 17 articles containing scales were found through citation checks of articles that used scales as outcome measures. Hence, 67 articles were read at this stage. Of these, 22 articles were excluded for various reasons (e.g., survey rather than validated instrument, instruments measuring concepts other than patient participation such as how patients cope with poor health). In total, 45 citations were included in the study (Figure 1).

An overview of the 45 instruments and their abbreviations included in this review is presented in Table 2.

### 3.1. Concepts

Based on the definitions of patient participation and its related concepts by Castro and colleagues, the concept of patient participation was represented most often in the included studies (*n* = 24), followed by patient centeredness (*n* = 12) and patient empowerment (*n* = 9) [32]. In terms of the patient-centeredness instruments, aspects such as the patients’ perceptions of informative, respectful, and attentive staff in the HSOPE [48] care coordination; continuity and shared responsibility in PPIC [80]; and physical comfort and emotional support in QPCC [86] were also measured.

Patient empowerment instruments contained intrapersonal and behavioral empowerment aspects, such as positive communication between patients and caregivers [47], illness and stress management [72], and encouragement to take control of one’s own health [78], as well as empowerment facilitators such as social support, access to staff, and information in PPPNBS [81].

Many of the patient participation instruments were intended to measure patients’ rights and opportunities to engage in planning and decision making about their care. CollaboRATE [43], iSHARE [52], a decision-making instrument used in surgical treatment [58], PPRQ [59], SDM-Q-9 [70], CAHPS [71], PPPIP [79], and 4Ps [82], all focused on sharing knowledge and patient participation in clinical care contexts. While PCPS [53] focused primarily on the patient communication pattern, other patient participation instruments, such as SURE [69], PPIQ [83], and PrepDM [85], focused on decisional conflict and patient readiness to make a decision. In addition, the evaluation of communication [44], decision regret [45], decision-making effectiveness [54] and the decisional balance of a patient’s choice in terms of substance abuse treatment was in focus [76].

### 3.2. Instruments including Environmental Aspects

No single instrument, item, or explanatory text was found to incorporate the physical environment. While most instruments had no context-driven items at all, 19 instruments considered the social environment to various extents. Of these instruments, few were theoretically grounded in a model that acknowledges the interplay between a person’s health and the environment for the purpose of enhancing patient participation (e.g., see [61]).

The instruments containing the most social environment items were generally categorized as patient-centered and often covered several domains. Aspects such as family and friend involvement were covered in CAHPS [71] and QPCCC [86], the resources available in the neighborhood to support patients in coping with health conditions were covered in IEXPAC [49] and PPIC [80], the extent to which healthcare providers ask about a patient’s family life was covered in PPPC-R [61], concerns about how to manage family life were covered in PCCE [63] and QPCCC [86], and adequate information about what to expect in terms of challenges in daily life was covered in the CPEQ [72].

Furthermore, a patient’s ability to cope with personal and social concerns and the degree to which she or he, at hospital discharge, was engaged in discussions regarding sexual activity, housework, gardening, and returning to work was considered [47,65].

Some instruments, such as DCS [46], primarily focused on patient support from others in making a health-related decision or receiving help in identifying people and/or resources to support in IEXPAC [49], PPIC [80], and PPPNBS [81]. Other instruments contained items asking the patient if his or her significant other had been informed and had the opportunity to be involved in PPRQ [59], CAHPS [71], and CTM [73]. In addition, a few instruments containing social environment aspects were designed for healthcare evaluation purposes. For example, Masters and colleagues [67] developed a questionnaire to measure whether specific processes or events occur following the transitions of older adults from the hospital to the home environment. In this instrument, quality entails that care is linked to the goals of patients, caregivers, and families and that these goals should also concern lifestyle, community participation, relationships, and emotional well-being.

### 3.3. Generic Instruments

Thirteen instruments were labeled as generic. Eleven of them measured patient participation [43,44,45,46,56,68,69,70,82,83,85]. One measured patient empowerment [78], and one measured patient centeredness [75]. Most of the generic instruments contained few items and focused on shared decision making. For example, CollaboRATE [43], SDM-Q [68], and the shorter version of SDM-Q9 [70] measured the effort of healthcare professionals toward supporting patient participation in care-related decisions.

When looking at the different generic instruments, one can see that they aim at measuring different facets of decision making. SURE [69] and PrepDM [85] measured patients’ readiness and perceptions of support to make decisions before the actual encounter between patients and healthcare professionals, while the decisional regret scale measured decisional regret after a care decision was already made [47]. There are also examples of generic instruments targeting decisional conflict before or after a decision has been made [46]. The other generic patient participation instruments included the 4Ps instrument, measuring patient preferences and whether they have been experienced in encounters with healthcare professionals [82]; PPIQ [83], measuring patients’ perceptions of communication, interest in their agenda, empathy, and patient involvement in care; and COMRADE [44], measuring risk communication and patients’ confidence in decisions. In addition to these generic instruments, PAM [78] measured patient activation in terms of patient knowledge and confidence, and CPS [75] focused on patients’ preferred roles in medical decision making.

### 3.4. Healthcare Continuum

#### 3.4.1. Instruments for Inpatient Care

Most instruments were intended to be used in inpatient care. Among them were instruments measuring patient participation during hospitalization; for example, the instruments of Arnetz [65] and He [51] were developed to be used in heart disease settings. These instruments focused on patient experiences involving nurses’ information, support, and recommendations. PPQ [60] was developed to measure patient participation in heart and lung disease decisions during inpatient care. IIMSS [50] focused on patients’ involvement in their own medical treatment with the aim of increasing patient safety. PPE-15 [84] determined a patient’s overall care experiences. PPPNBS [81] measured the process of empowerment in inpatient care. In addition, Heggland’s instrument was developed to assess patient participation in surgical wards [58].

The QPCCC instrument [86] was based on the quality standards of the Institute of Medicine (IOM) [1] and was developed for cancer patients. Additionally, PES-Q [55], iSHARE [52], and CPEQ [72] were developed to be used in oncology settings. The authors who created PES-Q and CPEQ stated that these instruments specifically measure patients’ empowerment strategies. DES-10 [77] measured patients’ engagement in decisions regarding cancer care. The CAHPS [71] measured overall quality of care with the inclusion of items measuring patients’ opportunities to participate and their shared decision making. PCCE [63] was a more general instrument that captured care quality broadly but focused on decision making and the information provided. PPED [66] was developed to measure participation in an emergency department.

The remaining instruments had more specific areas of use, such as for pressure injury prevention, i.e., PPPIP [79], as well as the prevention of chronic inflammatory arthritis [57], diabetes [47], and chronic pain [64]. These instruments did not specify if they targeted inpatient care; thus, they could also be used in other settings.

#### 3.4.2. Care Transition between Various Contexts

Two instruments specifically aimed to measure care transitions, i.e., between the inpatient setting and primary care or the home. For example, Master’s instrument [67] was developed to measure older adults’ experiences of transitional care and CTM [73] was developed to measure quality-of-care transitions from the patient perspective. Both instruments aimed to capture the experiences of the entire care transition phase and not only the hospital discharge phase. Questions in CTM concerned whether patients felt that they agreed on their care plan and understood their care plan and medication. IEXPAC [49] and PPIC [80] measured persons’ experiences of how care managers were able to integrate and coordinate care across professionals, facilities, and support systems and were able to remain constant over time and between visits. The instruments were patient- and family-centered and based on shared responsibility among patients, family members, and caregivers. Other instruments that measured care coordination were PCCCT [62] and CQ-index [74]. PCCCT captured patients’ experiences with the involved professionals, the degree of coordination by providers, and the degree of emphasis on patients’ problems, goals, and roles. The CQ index measured patients’ experiences in terms of collaboration between general practitioners and specialists.

#### 3.4.3. Outpatient Care

Other instruments were developed to be used in the outpatient context. PPRQ [59] aimed to measure patient participation in a rehabilitation context, and PPPC-R [61], HSOPE [48], and Small’s [54] instruments were developed to assess patient participation in outpatient settings. Other instruments, such as the one by Finnell and Lee [76], aimed to assess patient readiness for shared decision making in a substance abuse context. The CDIS [42] aimed to measure the clinical decision-making involvement and satisfaction of patients in mental healthcare.

## 4. Discussion

This paper reviewed the available instruments aiming to measure patient participation in healthcare with a specific focus on the related concepts and how environmental factors are considered. Given the lack of consensus regarding the definition of patient participation, our study followed Castro and colleagues [32] and attempted to treat the concept broadly and thus included other related concepts. As patient participation is an important quality measure of care and rehabilitation [3], we explored the instruments used throughout the healthcare system where the opportunity for patient participation is likely and where it can have an important impact on healthcare delivery and patient outcomes.

We identified many instruments that indicate that participation is an important area of interest for healthcare researchers, which was also shown by the wide international scope and range of contexts for which the instruments were developed. Given that it is a new field, it is not surprising that there are many development parallels, which could even be seen as a strength, as the combined components of this research help build a coherent construct theory. However, the large number of instruments raises questions about the clinical relevance of the vast number of instruments measuring similar domains. Many of these instruments were developed and tested for specific diagnoses or contexts but could very well be used in other areas. The instrument questions were often general and concerned with access to information or the opportunity for patients to share in decision making about their care and treatment. There seems to be a lack of knowledge transfer and agreement on how to best measure patient participation and researchers have continued to modify the existing instruments rather than developing new instruments that are suitable for the current healthcare context—for example, those suitable for considering the individual in his or her environment and as a part of society. Thus, in future research, the large content overlap could be used to guide a unified patient participation measurement scale.

The findings in this review confirm the lack of environmental focus among researchers and thus, the insufficient knowledge transfer to healthcare professionals and policymakers on how to embed contextual factors in patient participation and the inherent decision-making processes [87]. That is, despite the consensus that the environment plays a vital role in supporting patients, especially those with complex health conditions, to overcome their limitations following illness, there was no single item considering the physical environment in the instruments included in this review. We are not the first to highlight that the environment is important to consider in patient participation and person-centered care. As Kvæl and Bergland [88] concluded in their study on the environment’s influence on patient participation in the context of intermediate care of older people, the environment is not as recognized as it should be, even though it is a vital part of healthcare practice that mediates patient participation and supports person-centered care. 

We do not mean that all instruments, in all healthcare settings, should address the physical and/or social environment. The purpose of this review was to scope the field to see if any instrument entailed such aspects. We argue that for healthcare staff working in home and community settings, as well as those involved in prehospital discharge planning sessions, having a dialogue with patients that includes the environment is crucial. However, no such instrument seems to exist, which may inspire future research. This is important, as today the home environment is the main place for care and rehabilitation [23,33] and many decisions around the environment must be made. The home is very different from a hospital environment and can have physical (e.g., no elevator) and social (e.g., crowdedness, maintaining family life) characteristics that may be experienced as disabling. When a patient has complex care needs and is rehabilitated at home, the decision-making process cannot be simplified to consideration of a few alternatives. Their needs will change over time and the results may be affected by the environment, care provider, and context. As such, providers need to pay attention to and get involved in an iterative decision-making process that focuses also on daily life and considers the person’s physical and social environment. Earlier studies have shown that this is not the norm in clinical practice today [27]. In this review, we found one instrument by Masters and colleagues [67] that could serve as inspiration for future development of instruments in transitional as well as home and community care. Knowledge about patients’ experiences is key to improving care processes and their instrument contained a range of social-environmental aspects which measured the quality of care. 

The generic instruments in this review had only a few items and focused on different facets of shared decision making, but mainly focused on patients’ overall satisfaction with their care and the information provided. This finding is not surprising, as the focus traditionally has been on evaluating patients’ experiences with participation in care decisions. Thus, the current assessments of shared decision making imply a very limited perspective of person-centered care, where, in addition to the experience of patient participation in care decisions, other aspects must be included. Moreover, some instruments focused on a meeting with a single healthcare professional—e.g., a meeting between a medical doctor and a patient, which limited their use. Today, healthcare is increasingly delivered by multidisciplinary healthcare teams; therefore, future patient participation instruments would benefit from not being tied to a specific profession.

The scoping review method in this work was useful, as the topic was both complex and heterogeneous in nature and provided us with an overview of the available instruments for measuring patient participation across the continuum of care [8,14,89]. The scope was broader than those of previous overviews aiming to evaluate instrument reliability and validity (e.g., see [90,91]). One strength of this review was that it was conducted by researchers with different professional backgrounds (nursing, social work, neurology, rehabilitation medicine, and occupational therapy), all with experience working with patients in partnerships. Thus, based on the clinical experiences of working with patients with complex health needs across a continuum of care, we argued that for patient participation instruments to be relevant and usable, they should include aspects of the physical and social environments. 

### Limitations

This review has some limitations that must be considered in the interpretation of the results. We searched a limited selection of databases, and although we identified a large number of studies and instruments, we might have missed some relevant instruments. In addition, we did not include an analysis of how much the instruments have been used, as this was beyond the scope. There might be instruments that were included that reached only the development phase. We did not assess the psychometric properties of the included instruments which could be helpful for potential users. However, unlike a systematic review, the purpose of a scoping review is to give an overview of a potentially large and diverse body of relevant literature [37]. In such an overview, a wide range of research designs could be included, and the material might not necessarily be quality appraised. In addition, the inconsistency with which participation, as a concept, was presented across instruments meant that we were unable to synthesize the material in a qualified way; thus, this synthesis should be prioritized in future research.

## 5. Conclusions

This review shows that there are a large number of instruments that measure participation, but few that include the environment as an important factor in decision making concerning care and treatment. Our work is the first to study instruments from this perspective and shows the current research gap in this area, thus demonstrating the need for further research.

## Figures and Tables

**Table 1:** Overview of inclusion criteria.

Inclusion
Peer-reviewed journal articles
Published between 2009 and 2020
Participants: adults who are aged 18 years or over
The review considered any self-reported instruments that intended to measure patient participation in healthcare
**Exclusion**
Qualitative studies
Systematic reviews
Studies involving participants less than 18 years of age
Studies published in languages other than English
Not original research or research using a single item survey question
No instrument was available to in the article or attached as an appendix
The measure was designed to be completed by someone other than the patient
The paper described application of the measure but not its development

**Table 2:** Overview of instruments.

Name of Instrument	Abbreviation	Country of Origin	Concept ^a^	Type of Instrument ^b^	Care Context ^c^	Environmental Factors	Reference
Clinical Decision-making Involvement and Satisfaction	CDIS	UK	Patient participation	Specific	Outpatient care	^_^	[42]
CollaboRATE	CollaboRATE	USA	Patient participation	Generic	Generic	^_^	[43]
COMRADE	COMRADE	UK	Patient participation	Generic	Generic	^_^	[44]
Decision Regret Scale	N/A	Canada	Patient participation	Generic	Generic	^_^	[45]
Decisional Conflict Scale	DCS	Canada	Patient participation	Generic	Generic	Yes Social	[46]
Diabetes Empowerment Questionnaire	N/A	Iran	Patient empowerment	Specific	N/A Diabetes	Yes Social	[47]
Health Services OutPatient Experience questionnaire	HSOPE	Italy	Patient centeredness	Specific	Outpatient care	^_^	[48]
Instrument for Evaluation of the Experience of Chronic Patients	IEXPAC	Spain	Patient centeredness	Specific	Care transition	Yes Social	[49]
Inpatient Involvement in Medication Safety Scale	IIMSS	China	Patient empowerment	Specific	Inpatient care	^_^	[50]
Instrument measuring empowerment needs of patients after a percutaneous coronary intervention	N/A	China	Patient empowerment	Specific	Inpatient care	Yes Social	[51]
iSHARE patient questionnaires	iSHARE	The Netherlands	Patient participation	Specific	Inpatient care	^_^	[52]
Patient communication pattern scale	PCPS	Israel	Patient participation	Specific	N/A Patient – physician encounter	^_^	[53]
Patient empowerment in long-term conditions	N/A	UK	Patient empowerment	Specific	Outpatient care	Yes Social	[54]
Patient Empowerment Strategies Questionnaire	PES-Q	Greece	Patient empowerment	Specific	Inpatient care	Yes Social	[55]
Patient Engagement Index	PEI	China	Patient participation	Generic	Generic	^_^	[56]
Patient Motivation Questionnaire	N/A	Egypt	Patient empowerment	Specific	Inpatient care	^_^	[57]
Patient participation in decision making in surgical treatment	N/A	Norway	Patient participation	Specific	Inpatient care	^_^	[58]
Patient Participation in Rehabilitation Questionnaire	PPRQ	Sweden	Patient participation	Specific	Outpatient care	Yes Social	[59]
Patient Participation Questionnaire	PPQ	Denmark	Patient participation	Specific	Inpatient care	^_^	[60]
Patient Perception of Patient-Centeredness Questionnaire	PPPC-R	Canada	Patient centeredness	Specific	Outpatient care	Yes Social	[61]
Patient-centered coordination by a care Team questionnaire	PCCCT	Canada	Patient centeredness	Specific	Care transition	^_^	[62]
Patients and the Cancer Care Experience Survey	PCCE	USA	Patient centeredness	Specific	Inpatient care	Yes Social	[63]
Patients’ Perceived Involvement in Care Scale	M-PICS	USA	Patient participation	Specific	N/A Chronic pain	^_^	[64]
Questionnaire for measuring patient views of involvement in myocardial infarction care	N/A	Sweden	Patient centeredness	Specific	Inpatient care	Yes Social	[65]
Questionnaire for patient participation in emergency departments	PPED	Sweden	Patient participation	Specific	Inpatient care	^_^	[66]
Questionnaire to measure older people’s experience of the Transition Care Program	N/A	Australia	Patient centeredness	Specific	Care transition	Yes Social	[67]
Shared decision-making questionnaire	SDM-Q	Germany	Patient participation	Generic	Generic	^_^	[68]
Sure of myself; Understand information; Risk-benefit ratio; Encouragement	SURE	Canada	Patient participation	Generic	Generic	_	[69]
The 9-item Shared Decision Making Questionnaire	SDM-Q-9	Germany	Patient participation	Generic	Generic	^_^	[70]
The CAHPS Cancer Care Survey	CAHPS	USA	Patient participation	Specific	Inpatient care	Yes Social	[71]
The Cancer Patient Empowerment Questionnaire	CPEQ	Denmark	Patient empowerment	Specific	Inpatient care	Yes Social	[72]
The Care Transition Measure	CTM	USA	Patient centeredness	Specific	Care transition	Yes Social	[73]
The Consumer Quality Index (CQ-index) Continuum of Care	CQ-index	The Netherlands	Patient participation	Specific	Care transition	^_^	[74]
The Control Preferences Scale	CPS	Canada	Patient centeredness	Generic	Generic	^_^	[75]
The Decisional Balance for Patient Choice in Substance Abuse Treatment	N/A	USA	Patient participation	Specific	Outpatient care	Yes Social	[76]
The Decisional Engagement Scale	DES-10	USA	Patient participation	Specific	Inpatient care	^_^	[77]
The patient activation measure	PAM	USA	Patient empowerment	Generic	Generic	^_^	[78]
The Patient Participation in Pressure injury Prevention scale	PPPIP	Australia	Patient participation	Specific	N/A Pressure injury	^_^	[79]
The Patient Perceptions of Integrated Care survey	PPIC	USA	Patient centeredness	Specific	Care transition	Yes Social	[80]
The Patient Perceptions of Patient-Empowering Nurse Behaviours Scale	PPPNBS	USA	Patient empowerment	Specific	Inpatient care	Yes Social	[81]
The Patient Preferences for Patient Participation tool	The 4Ps	Sweden	Patient participation	Generic	Generic	^_^	[82]
The Patient-Professional Interaction Questionnaire	PPIQ	Italy	Patient participation	Generic	Generic	^_^	[83]
The Picker Patient Experience Questionnaire	PPE-15	UK	Patient centeredness	Specific	Inpatient care	Yes Social	[84]
The Preparation for Decision Making scale	PrepDM	Canada	Patient participation	Generic	Generic	^_^	[85]
The Quality of Patient-Centered Cancer Care	QPCCC	Australia	Patient centeredness	Specific	Inpatient care	Yes Social	[86]

^a^ According to Castro et al [32], ^b,c^ Generic, i.e., instruments that could be used for different populations and contexts, or specific, i.e., instruments that could be tested and used in a specific care context or for a specific diagnosis.

## Data Availability

All data relevant to the study are included in the article or uploaded as Supplemental Information.

## Supplementary Materials

The following are available online at https://www.mdpi.com/article/10.3390/ijerph19042003/s1, Figure S1: Shows the tailored search query of each database and follow-up search of the databases.
