# Supplementary material for: Patient Participation and the Environment: A Scoping Review of Instruments
Source: Int J Environ Res Public Health. 2022 Feb 11;19(4):2003. doi: 10.3390/ijerph19042003 (PMC8872044; doi:10.3390/ijerph19042003)
Supplement: Supplementary file 1 [file ijerph-19-02003-s001.zip › ijerph-1535799-supplementary.pdf]

Supplementary Materials: Searches

Cinahl, 20190902

S1	TI ("patient empowerment" OR "patient participation" OR "patient engagement" OR "patient involvement" OR "Client Participation" OR "shared decision making" OR "shared decision-making" OR "continuum of care" OR "patient centered care") OR AB ("patient empowerment" OR "patient participation" OR "patient engagement" OR "patient involvement" OR "Client Participation" OR "shared decision making" OR "shared decision-making" OR "continuum of care" OR "patient centered care")		12,251
S2	TI ((measurement OR assessment OR scale OR scales OR instrument OR score OR scores) OR (questionnaire OR survey OR validation OR psychometrics)) OR AB ((measurement OR assessment OR scale OR scales OR instrument OR score OR scores) OR (questionnaire OR survey OR validation OR psychometrics))		956,982
S3	S1 AND S2		3,951
S4	S1 AND S2	Limiters - Published Date: 20090101-20191231 , Academic Journals, English	2,261

PsycInfo, 20190902

S1	TI ("patient empowerment" OR "patient participation" OR "patient engagement" OR "patient involvement" OR "Client Participation" OR "shared decision making" OR "shared decision-making" OR "continuum of care" OR "patient centered care") OR AB ("patient empowerment" OR "patient participation" OR "patient engagement" OR "patient involvement" OR "Client Participation" OR "shared decision making" OR "shared decision-making" OR "continuum of care" OR "patient centered care")		6,397
S2	TI ((measurement OR assessment OR scale OR scales OR instrument OR score OR scores) OR (questionnaire OR survey OR validation OR psychometrics)) OR AB ((measurement OR assessment OR scale OR scales OR instrument OR score OR scores) OR (questionnaire OR survey OR validation OR psychometrics))		1,210,988
S3	S1 AND S2		2,363
S4	S1 AND S2	Limiters - Published Date: 20090101-20191231 , Academic	1,471

		Journals, English	

PubMed, 20190902

#1 ("patient empowerment"[Title/Abstract] OR "patient participation"[Title/Abstract] OR "patient engagement"[Title/Abstract] OR "patient involvement"[Title/Abstract] OR "Client Participation"[Title/Abstract] OR "shared decision making"[Title/Abstract] OR "shared decision-making"[Title/Abstract] OR "continuum of care"[Title/Abstract] OR "patient centered care"[Title/Abstract]) 20,488

((measurement[Title] OR assessment[Title] OR scale[Title] OR scales[Title] OR instrument[Title] OR score[Title] OR scores[Title] OR questionnaire[Title] OR survey[Title] OR validation[Title] OR psychometrics[Title]))) 685,871

#3 #1 AND #2 1,297

#4 Filters: Journal Article; Publication date from 2009/01/01; English 1,070

Number of hits in all databases 4,802

Hits after automated deduplication in EndNote 4,116

Updated search, 2020-06-12

Cinahl

S1	TI ("patient empowerment" OR "patient participation" OR "patient engagement" OR "patient involvement" OR "Client Participation" OR "shared decision making" OR "shared decision-making" OR "continuum of care" OR "patient centered care") OR AB ("patient empowerment" OR "patient participation" OR "patient engagement" OR "patient involvement" OR "Client Participation" OR "shared decision making" OR "shared decision-making" OR "continuum of care" OR "patient centered care")		15,610
S2	TI ((measurement OR assessment OR scale OR scales OR instrument OR score OR scores) OR (questionnaire OR survey OR validation OR psychometrics)) OR AB ((measurement OR assessment OR scale OR scales OR instrument OR score OR scores) OR (questionnaire OR survey OR validation OR psychometrics))		1,200,929
S3	S1 AND S2		5,004
S4	S1 AND S2	Limiters - Published Date: 20190701-20201231 , Academic Journals, English	604

PsycInfo

S1	TI ("patient empowerment" OR "patient participation" OR "patient engagement" OR "patient involvement" OR "Client Participation" OR "shared decision making" OR "shared decision-making" OR "continuum of care" OR "patient centered care") OR AB ("patient empowerment" OR "patient participation" OR "patient engagement" OR "patient involvement" OR "Client Participation" OR "shared decision making" OR "shared decision-making" OR "continuum of care" OR "patient centered care")		6,866
S2	TI ((measurement OR assessment OR scale OR scales OR instrument OR score OR scores) OR (questionnaire OR survey OR validation OR psychometrics)) OR AB ((measurement OR assessment OR scale OR scales OR instrument OR score OR scores) OR (questionnaire OR survey OR validation OR psychometrics))		1,257,413
S3	S1 AND S2		2,561
S4	S1 AND S2	Limiters - Published Date: 20190701-20201231 ,	147

		Academic Journals, English	

PubMed

#1 ("patient empowerment"[Title/Abstract] OR "patient participation"[Title/Abstract] OR "patient engagement"[Title/Abstract] OR "patient involvement"[Title/Abstract] OR "Client Participation"[Title/Abstract] OR "shared decision making"[Title/Abstract] OR "shared decision-making"[Title/Abstract] OR "continuum of care"[Title/Abstract] OR "patient centered care"[Title/Abstract])	23,118
((measurement[Title] OR assessment[Title] OR scale[Title] OR scales[Title] OR instrument[Title] OR score[Title] OR scores[Title] OR questionnaire[Title] OR survey[Title] OR validation[Title] OR psychometrics[Title])))	723,558
#3 #1 AND #2	1,488
#4 Filters: Journal Article; Publication date from 2019/07/01; English	258
Number of hits in all databases	1,009
**Hits after deduplication in EndNote**	**811**
